# Decentralized Cooperative TOA/AOA Target Tracking for Hierarchical Wireless Sensor Networks

**DOI:** 10.3390/s121115308

**Published:** 2012-11-08

**Authors:** Ying-Chih Chen, Chih-Yu Wen

**Affiliations:** Department of Electrical Engineering, Graduate Institute of Communication Engineering, National Chung Hsing University, Taichung 402, Taiwan; E-Mail: chenyingchr@gmail.com

**Keywords:** wireless sensor networks, target tracking, Bayesian filtering, neural networking, covariance intersection

## Abstract

This paper proposes a distributed method for cooperative target tracking in hierarchical wireless sensor networks. The concept of leader-based information processing is conducted to achieve object positioning, considering a cluster-based network topology. Random timers and local information are applied to adaptively select a sub-cluster for the localization task. The proposed energy-efficient tracking algorithm allows each sub-cluster member to locally estimate the target position with a Bayesian filtering framework and a neural networking model, and further performs estimation fusion in the leader node with the covariance intersection algorithm. This paper evaluates the merits and trade-offs of the protocol design towards developing more efficient and practical algorithms for object position estimation.

## 1. Introduction

Giving the limited power and processing capability in a sensor mote, a critical challenge of target tracking is how to acquire suitable data and perform information processing at the local level through cooperative communication and networking in the vicinity of the target. Thus, scalability and the need to conserve energy lead to the idea of hierarchically organizing the sensors, which can represent the target state and incorporate statistical models for the sensing schedule and target positioning. This paper aims to develop a fully distributed method for cooperative target tracking in wireless sensor networks from two perspectives: (1) energy-balanced tracking and (2) improved estimation accuracy.

The first perspective is to build up an energy-balanced tracking network architecture. In this work, the concept of leader-based information processing is conducted to automatically achieve cooperative sensor scheduling with multiple tasking sensors in a cluster-based network topology based on sensor residual energy level, target information, and estimation quality. To avoid the ambiguity, the clusterhead and the cluster members refer to the original network topology, whereas the leader and the sub-cluster members refer to the sensor group for the tracking task. Random timers and local criteria are used to determine the tracking responsibility of the clusters. Afterwards, a sub-cluster of the corresponding cluster for the tracking task is formed by a leader, which can be a clusterhead or a cluster member in the original cluster-based network. The second perspective is to explore the behaviors/characteristics of a target such that supplementary information can be applied to improve estimation accuracy. Within the sub-cluster, the sensing nodes provide their measurements to the leader. Upon receiving the measurements, the leader fuses the local estimates from the sub-cluster members and reports it to the clusterhead. When the target moves out the region of the current active sub-cluster, the leader needs to trigger the leader handoff procedure (detailed in Section 3.4).

As shown in [1], compared with the dynamic clustering approach in a flat network topology, the static clustering approach incurs a larger location error since a clusterhead may not be a good local controller for estimating the location and reporting the event due to target movement. However, given a fixed hierarchical network topology, dynamic clustering approaches may not be feasible. Therefore, considering a cluster-based network topology, we introduce a distributed cooperative target tracking system, *Two-level Clustering Approach via Timer* (TCAT), which aims to improve the energy efficiency and provide good estimation accuracy. Here level-one clustering indicates the original network topology with control of clusterheads. Level-two clustering means the sub-cluster formation for the tracking task with control of tasking leaders. Therefore, the information flow goes through the sub-cluster members to the leader, and then to the clusterhead, and vice versa. Accordingly, the TCAT scheme performs target localization in four phases: (I) Tasking Leader Selection; (II) Choosing the Sub-Cluster Members; (III) Target Positioning; and (IV) Sub-Cluster Member Reselection and Leader Handoff.

In Phases I and II, random timers and local information are applied to adaptively select a tasking leader and sub-cluster members for the localization task. In Phase III, the Bayesian particle filter [2,3] is used to estimate the unknown target position from state equations. The objective is to find feasible position to minimize the error of the state vector. After obtaining the initial position estimate, the localization adjustment problem can be solved by applying a neural networking model, which focuses on improving positioning accuracy. Afterwards, the covariance intersection algorithm [4] is adopted to perform estimation fusion. In order to maintain tracking stability, Phase IV performs the leader handoff task.

The major contributions and key features of this paper are listed as follows. (1) We propose a novel cooperative positioning approach. (2) One of the main advantages of Bayesian framework is that the tasking sensor carries along a complete distribution of estimates of the target position. Thus, the distribution is inherently a measure of the accuracy of the positioning system. (3) Due to the characteristics of the cooperative information processing, the proposed estimation fusion approach owns adaptive flexibility when dealing with uncertainty in position estimation. (4) In practice, two basic approaches can be applied to conserve power in a sensor network, either by the power management for sensor sleeping or by the design of low-power operation hardware. Our scheme is actually a distributed scheduling strategy for target tracking. The concept of “power management for sensor sleeping” is applied to select a subset of sensors for exploring detailed target information. The selected sub-cluster members (active nodes) are performing the tracking task and the inactive nodes can go to the idle/sleep mode. (5) We compare and contrast the existing tracking approaches with the proposed scheme. (6) We outline the technical foundations of the tracking techniques and present the tradeoffs in the algorithm design.

The rest of the paper is organized as follows. Section 2 briefly reviews the literatures on target tracking. Section 3 formulates the problem, derives a distributed solution, and describes a neural networking refinement model for target positioning. Section 4 presents an estimation-theoretic analysis of the proposed mechanism to assess the achievable estimation accuracy. Then, Section 5 considers the energy consumption of the proposed tracking scheme. In Section 6, the feasibility of the proposed scheme is examined via simulation. The performance comparison between the proposed approach and the scheme with a hierarchical network topology in [5] is presented. Section 7 makes a conclusion and shows future research directions.

## 2. Literature Review

There are five major categories for the target tracking solutions [1]: tree-based tracking, cluster-based tracking, prediction-based tracking, mobicast message-based tracking, and hybrid tracking. Studies have shown that the cluster-based tracking algorithms have better network scalability and resource utilization compared with those in other categories. Prediction-based tracking rely on tree-based and cluster-based tracking in addition to the prediction method, but the tracking accuracy cannot be guaranteed. Mobicast message-based tracking method depends on prediction, which is a multi-cast method in which message is delivered to a group of nodes that change with time according to estimated velocity of moving entity. Scheduling strategies vary in target tracking protocols and time synchronization may be needed to set the wake up and sleep timings of sensor nodes.

Since the proposed approaches fall into the category of cluster-based tracking, we focus on the research results of this category. The following subsections briefly describe the current literature of target tracking with respect to the number of tasking sensors.

### 2.1. Single Tasking Sensor

In the current literature, the general problem formation of target tracking is reformed to be a sensor selection problem with the uniform sampling interval and without incorporating the target dynamics, *i.e.*, the information-driven sensor query (IDSQ) approach [6] and the entropy approach based on sensor selection [7]. In contrast, the authors in [8,9] propose adaptive scheduling strategies to choose the single tasking sensor and determine the sampling interval simultaneously. In [8], the sensors are scheduled in two tracking modes: (1) the fast tracking approaching mode with the unsatisfactory predicted tracking accuracy; and (2) the tracking maintenance mode with the satisfactory predicted tracking accuracy. The approach employs an Extended Kalman Filter (EKF) based estimation technique to predict the tracking accuracy, and adopts a linear energy model to predict the energy consumption. In [9], the proposed algorithm applies the interactive multiple model (IMM) filter to estimate and predict the target's dynamic state and select the tasking sensor node and sampling interval for each time step based on both the tracking accuracy and the energy cost. The simulation results show that the proposed approach outperforms the popular extended Kalman filter (EKF) based tracking scheme for maneuvering target in terms of tracking accuracy and energy efficiency. In [10], a small region is specified in order to select the single tasking sensor for achieving energy conservation. The distributed IMM filter is employed to estimate target position and velocity. A novel dynamic grouping idea is proposed to schedule next tasking node. However, the IMM filter has to face the problem of high complexity, especially with the operation of dynamic grouping.

In general, the localization problem can be solved by the joint time-of-arrival (TOA)/angle-of-arrival (AOA) positioning scheme using a single seed (*i.e*., a sensor node with a known position). However, in the case of poor observations, more TOA/AOA measurements (*i.e*., multiple tasking sensors) may be applied to complement the measurements of the environment.

### 2.2. Multiple Tasking Sensors

For the purpose of increasing estimation accuracy and reliability, multiple tasking sensors may be scheduled to track the target with detection uncertainties. In [5], target localization strategies based on a communication protocol between the clusterhead and cluster members are presented. In these approaches, a subset of sensor nodes is selected for querying detailed target information. Although energy and communication bandwidth are conserved in a certain amount, the processing burden all falls on the clusterhead, which may drain its power quickly. Suganya, *et al.* [11] focuses on tracking error and energy management involved in tracking the target. In this approach, the sensor nodes collectively monitor and track the movement of the target, which involves detecting, clustering and localization of target.

In [12], an information-driven approach in ad hoc sensor networks is proposed to determine the tasking sensors in a “sensor collaboration” by dynamically optimizing the information utility of data for a given cost of communication and computation. In [13], a distributed estimation method is proposed based on mobile agent (MA) computing paradigm and generic sequential Bayesian filtering for the target state estimation at each time step. Nonetheless, the MA migration planning problem needs to be handled in order to achieve the desired tracking accuracy. The tracking schemes in [14,15] combine the mechanisms of the tree-based and cluster-based schemes and propose information-based target tracking methods. However, the proposed sensor systems still have to deal with complexity issues. Authors in [16–21] propose multi-sensor scheduling schemes for maneuvering target tracking in sensor networks, while not considering the motion information of the target. Williams, *et al.* [22] presents an integrated approach to dynamically routing measurements and models in a sensor network and examines the problem of tracking objects within a region wherein the responsibility for combining measurements and updating a posterior state distribution is assigned to a single sensor at any given time step. The proposed approach is able to substantially reduce the communications cost incurred in tracking an object. However, this strength comes along with the additional complexity of transmitting the representation of the state distribution between the tasking sensors. Comprehensive surveys of design challenges and recently proposed target tracking algorithms can be found in [1].

Note that most of these design approaches are dynamic clustering protocols in a flat network. In contrast, the method in [5] is built upon a static cluster-based network topology. Thus, reference [5] may provide a good way to benchmark the performance of the proposed tracking scheme.

## 3. Distributed Target Tracking Systems

**Figure 1.**
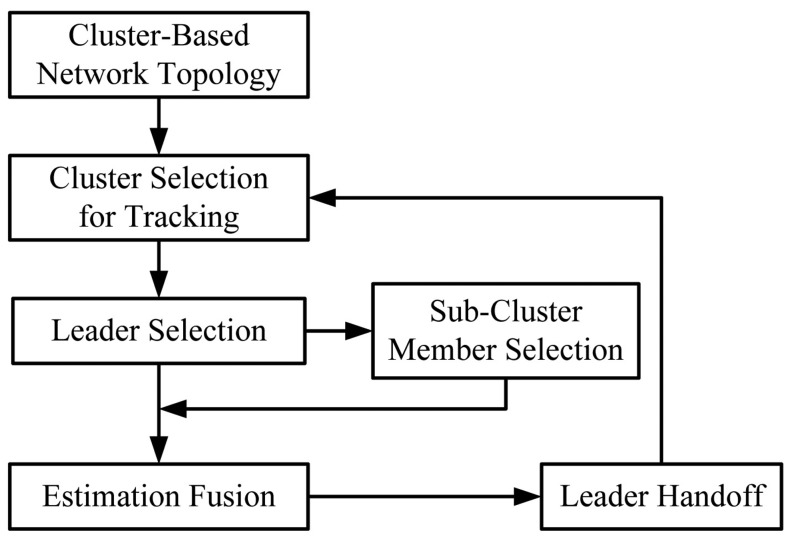
Illustration of block diagram for the TCAT method.

In this section, we present the proposed distributed cooperative target tracking system: *Two-level Clustering Approach via Timer* (TCAT) in a cluster-based network topology, which organizes the tracking task in four phases: tasking leader selection, choosing the sub-cluster members, target positioning, and sub-cluster member reselection and leader handoff. Therefore, the proposed tracking approach organizes a sub-cluster for the tracking task, allows each sub-cluster member to locally compute the target position, and uses cooperation to obtain the fused estimate in the leader node. The local level estimate of a sub-cluster member and the global level estimate of a leader can be derived by a Bayesian and neural networking framework, and the covariance intersection algorithm, respectively. The main assumptions are: (i) all sensors are homogeneous; (ii) the sensors are in fixed and known location; (iii) a pre-specified sub-cluster size *n* is applied to perform cooperative target positioning with angle-of-arrival (AOA) information or hybrid time-of-arrival/angle-of-arrival (TOA/AOA) information, (iv) the target periodically broadcasts a message for measurement purpose. Note that these assumptions may be applied to healthcare scenarios or habitat monitoring to locate patients or animals. The distributed tracking architecture in a cluster-based network is depicted in Figure 1.

### 3.1. Phase I: Tasking Leader Selection

**Figure 2.**
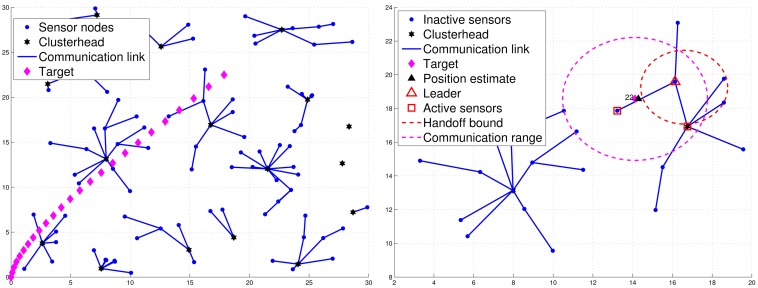
The cluster-based network topology and target movement with 25 time steps (**left**); an example of leader and sub-cluster member selection (**right**).

When sensors are first deployed, they may apply the CAWT [23] to establish the cluster-based network architecture (Figure 2(left)). However, due to the target movement, the clusterhead may not be a proper local controller in the neighborhood of the target. Thus, a cluster member may be a good control candidate and can be a leader for the tracking task. Denote a sensor with tracking responsibility as an active sensor. Otherwise, a sensor is marked as an inactive sensor. Suppose each sensor is an inactive sensor with the initial deployment. The tracking task is triggered when the target broadcasts a message of *L_id_*, where *L_id_* is a leader ID with an initial value zero, which is used to inform the active sensors to compete for being a leader of the tracking task. Thus, when sensor *i* receives the message sent from the target, it will broadcast a *Hello* message and become an active sensor, which allows itself to estimate how many neighboring active sensors it has. A *Hello* message consists of (1) the sensor ID of the sending sensor, (2) the leader ID of the sending sensor, and (3) the cluster ID of the sending sensor. Therefore, the sensors update their neighboring information (*i.e*., a counter specifying how many neighboring active sensors it has detected) and decrease the random leader waiting time (LWT) through the received Hello message sent from neighboring active sensors.

Assume the initial value of the waiting time of sensor *i*, ${\text{LWT}}_{i}^{\left(0\right)}$, is a sample from the distribution *U*(*C*, *D*′), where *D*′ = *C* + *D*, *C* and *D* are positive numbers, and *U*(·, ·) is a uniform distribution. The update formula for the random LWT is given by

(1) $${\text{LWT}}_{i}^{\left(j+1\right)}=\alpha \cdot {\text{LWT}}_{i}^{\left(j\right)}$$

where ${\text{LWT}}_{i}^{\left(j\right)}$ is the random LWT of sensor *i* at time step *j*, and 0 < *α* < 1. Note that the setting of random LWT may depend on sensor residual energy level, target information, and measurement quality (e.g., the channel condition, the accuracy of positioning system). When the timer of sensor *i* expires, it then broadcasts a *Leader* message to claim that it is leader *i* (e.g., *L_id_* = *i*) for the tracking task.

### 3.2. Phase II: Choosing the Sub-Cluster Members

Based on the claimed message sent from the leader and the cluster ID of the leader, the target will send a message to inform the active sensors about the corresponding cluster for the tracking task, which also notifies the active sensors with the same cluster ID to be the candidate sub-cluster members associated with the leader. To choose the members associated with a leader, instead of directly selecting the active sensors from the leader, the sensor selection may be determined based on the reporting order of target position estimates from the neighboring active sensors of the leader. Accordingly, a candidate sub-cluster member, say sensor *m*, may decrease its LWT along with an extra backoff time *BT_m_*, which is inversely proportional to the estimation accuracy, for reporting the estimate of target position. When the timer *LWT_m_* expires, sensor *m* will deliver the tracking information to leader *i*. That is, based on the time stamps of the received estimates, the target tracking group is then automatically formed with the leader.

For those active sensors without receiving a *Leader* message, they transmit the estimated target position directly to the clusterhead and become supplementary sub-cluster members. This is attributed to the fact that the active sensors may not have direct communication with the leader. Hence, they may send their tracking estimates to the clusterhead for providing supplementary information on the tracking task. Therefore, when the number of sub-cluster members meets the desired number *n*, the leader will perform the CI model (as detailed in Section 3.3.3) to obtain a global target position estimate and send a *Position* message to the clusterhead, which also serves to specify the final sub-cluster members for cooperative target tracking. If the leader does not collect sufficient number of estimates (*i.e*., |*M*| < *n*, M is the index set of the sub-cluster), then the leader may send its fused estimate to the clusterhead and request the clusterhead to incorporate the supplementary estimates if possible. Afterwards, the clusterhead will perform the CI algorithm based on the received fused estimate and the supplementary estimates. Note that in order to spread the energy burden in the network, the cluster is responsible for informing the base station about the target tracking and positioning. Figure 2(right) shows an example of leader and sub-cluster member selection.

### 3.3. Phase III: Target Positioning

This phase presents a measurement mechanism to estimate the target position. The localization operation is performed in three phases: “initial geometrical positioning”, “position estimation refinement”, and “estimation fusion”. For each sub-cluster member, the Bayesian particle filter is used to obtain an initial position estimate. Next, the localization adjustment problem can be solved by applying a neural networking model to refine the sample area and then redoing the particle filter to improve the positioning accuracy. Finally, estimation fusion is performed by the leader node in order to obtain a global estimate.

#### 3.3.1. Geometrical Positioning with Particle Filtering

The Bayesian particle filter [2] method may be preferred for object positioning because it is robust to noisy measurements, it allows for flexible information transmission, and it can be robust to lost or lossy data. Particle filter is an algorithm of estimation used to estimate the unknown target position from state equations. The objective is to find feasible position to make the error of state vector *x* minimum. The state vector is represented as a set of random samples updated and propagated with the algorithm. One of the main advantages of this approach is that the tasking sensor carries along a complete distribution of estimates of the target position. Therefore, the distribution is inherently a measure of the accuracy of the positioning system. If a given task requires certain accuracy, it is possible to determine if that level of accuracy is currently available. Therefore, our approaches may be computationally affordable by sensor nodes.

**Figure 3.**
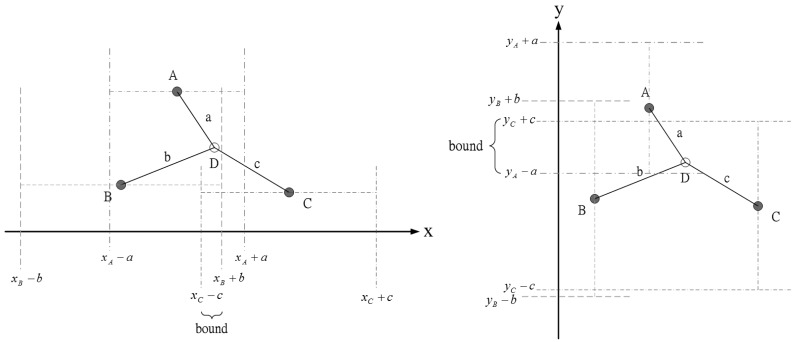
An example of obtaining the *x* and *y* coordinate bounds of the unknown sensor D by the distance and position information of the known sensors A, B, and C.

The idea in [24], using known sensor positions and the bounding-box algorithm to extrapolate the unknown target position, inspires us to choose a proper prior density for generating initial samples. Figure 3 shows an example how the measurement information (e.g., distance information) can be used to obtain the *x* and *y* coordinate bounds of the unknown target. Therefore, the unknown target combines its bounds on the coordinates to form a bounding box, which provides a good set of initial samples for the particle filtering. In this work, each sub-cluster member uses Bayesian particle filter to estimate the unknown target position and performs target positioning with angle-of-arrival (AOA) information or hybrid time-of-arrival/angle-of-arrival (TOA/AOA) information. The particle filter method is shown in Table 1.

#### 3.3.2. Position Estimation Refinement

Since the AOA measurement quality highly depends on the communication environment, this subsection presents estimation refinement criteria based on noisy AOA information, TOA information, and an angle-of-arrival neural networking (ANN) model. The purpose of the ANN model is to coordinate the initial target position estimate, the initial sample space, and the measurement information in a scenario with multiple tasking sensors such that effectively adjustment of angle information and a better sample area for particle filtering can be provided.

##### Angle-of-Arrival Neural Networking with CFBP

The feed-forward backpropagation (FFBP) and cascade-forward backpropagation (CFBP) are supervised learning algorithms for artificial neural networks which most commonly used for prediction, pattern recognition, and nonlinear function fitting [25]. Since the CFBP provides a better performance in terms of convergence time, optimum of network structure and recognition performance [26], the neural network with CFBP is applied for analyzing the performance of the TCAT

Assume that the network under consideration has a general architecture with three layers of neurons. In our case, input and output layer neurons are linear, whereas neurons in the hidden layer are tan-sigmoidal. Let the vector pairs in 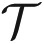 be sample representation of the unknown function *f* : ℛ*^n^* → ℛ*^p^*: $T={\left\{(X_{q},D_{q})\right\}}_{q=1}^{Q}$, where *n* is the neuron index range in the input layer, *p* is the neuron index range in the output layer, *X_q_* ∈ ℛ*^n^*, *D_q_* ∈ ℛ*^p^*, *Q* is the number of training vector pairs, and *q* is the iteration index. Note that *D_q_* is the desired vector response for the network input *X_q_*. Thus, the mean square error of the entire training set is: $ℰ=\frac{1}{Q}\sum_{q=1}^{Q}{ℰ}_{q}$, where ${ℰ}_{q}=\frac{1}{2}{\text{E}}_{q}^{T}E_{q}$, and *E_q_* is the instantaneous error of the training pair (*X_q_*, *D_q_*).

##### Estimation Adjustment

In order to adjust angle information, the three-layer perceptron neural network is considered. Based on the proposed network architecture, the goal of training is to maximize the correlation *C* between the signal of the hidden neuron and the residual output error [27]:

(2) $$C=\sum_{j=1}^{p}\left|\sum_{q=1}^{Q}\left(S({\text{z}}_{h}^{q})-S_{av})(\delta_{j}^{q}-\Delta_{j}\right)\right|$$

where 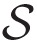
(·) is the signal function, $S_{av}=\frac{1}{Q}\sum_{q=1}^{Q}S({\text{z}}_{h}^{q}),\Delta_{j}=\frac{1}{Q}\sum_{q=1}^{Q}\delta_{j}^{q},{\text{z}}_{h}^{q}$ is the signal of the hidden neuron *h* in response to input pattern *X_q_*, $\delta_{j}^{q}$ is the familiar scaled output error at neuron *j*, Δ*_j_* is the average scaled error on the entire pattern set. Accordingly, for selecting the network parameters (weights and biases) that best approximate a given function, the backpropagation learning algorithm is considered to minimize the mean square error performance ℰ.

**Figure 4.**
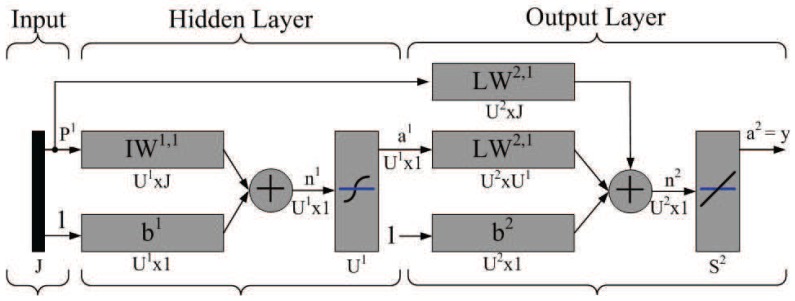
The three-layer perception network architecture for analyzing the performance of the TCAT.

**Figure 5.**
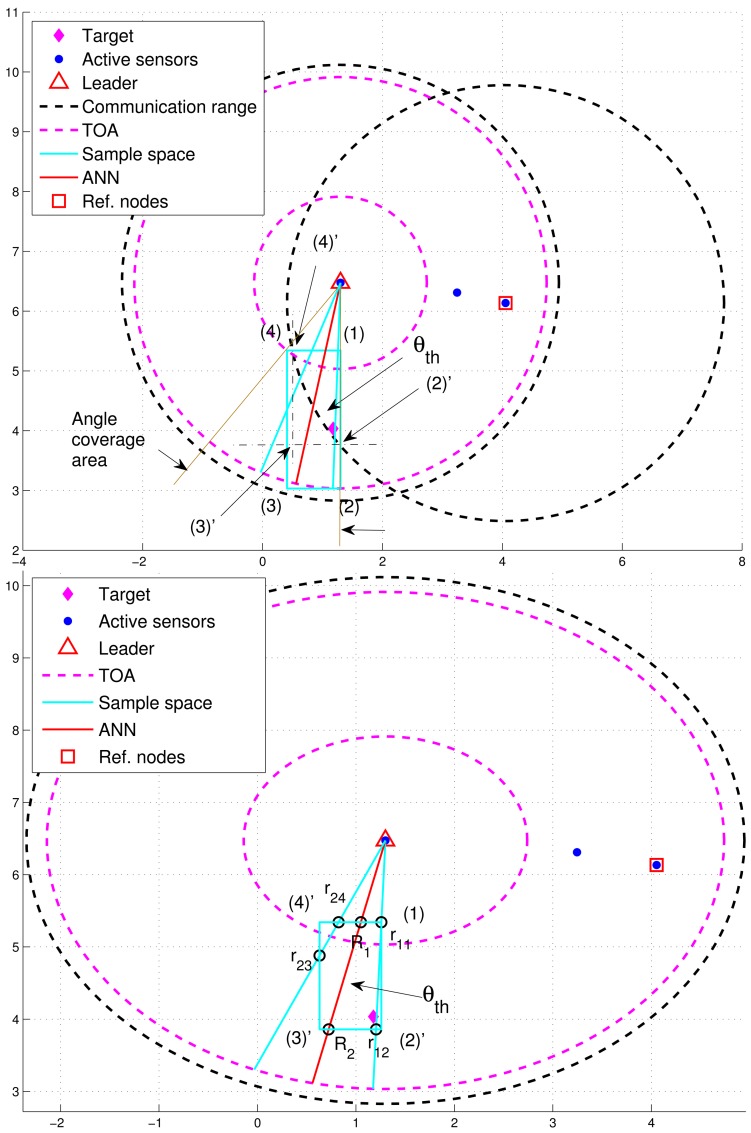
The initial sample area (**top**); the refined bounding box based on the reference angle information (**bottom)**.

Figure 4 illustrates the perception network architecture. Note that *J* represents the number of input neurons, which may denote the number of received messages from neighboring sensors, the AOA measurement of the estimated target, the desired value of sub-cluster size *n*, and the minimum angle coverage area of a tasking sensor with a right-hand-side angle boundary and a left-hand-side angle boundary (two brown lines as shown in Figure 5(top)), where the initial sample area is located within the coverage area. *U*^1^ denotes the number of hidden neurons. In the output layer, *U*^2^ represents the number of neurons, which may denote the network approximation results. Moreover, let IW and LW be the input weight matrix and layer weight matrix for the hidden layer and the output layer, respectively. Let *b*^1^ and *b*^2^ be bias vectors for the hidden layer and the output layer, respectively. Established upon the developed neuron network model, the behavior of the TCAT scheme may be abstracted with sensible settings, which is further discussed in Section 6.

##### Estimation Refinement with ANN

In order to improve positioning accuracy with particle filtering, one option is to determine an appropriate sample space for generating particles. As shown in Figure 5(top), the initial sample area is the rectangle defined by blue lines and four corners: (1), (2), (3), and (4) ordered clockwise, which serves as a basis for estimation refinement. Observe that the target is located within the defined rectangle and the pink “TOA” circles represent the minimum and maximum range estimates.

After importing the information of the defined rectangle and the tasking sub-cluster to the trained neural networking model, a reference angle information (RAI), (*i.e*., the angle information label with a red line), is proposed to refine the initial sample area for the prior density in Step 1 of Table 1. Without loss of generality, a tasking leader is applied as an example to describe the refinement procedures. With the RAI produced by the neural networking model, a threshold *θ_th_* (*i.e.*, the angle between the red line and the blue line in Figure 5) is used to adjust the initial sample area. Given the information of transmission range and the position knowledge of sub-cluster members, the leader node can choose a sub-cluster member, which is an active sensor and has a minimum overlapping area between the communication coverage and the initial sample area, as a reference node to narrow down the sample area. This is attributed to the fact that the position information and the communication coverage of a reference node can be applied to help reshape the initial sample area for localizing the target. On the basis of the RAI (the red line), denote *r*_1_*_i_* (*r*_2_*_j_*) as the intersection between the initial sample area and the right-hand-side angle boundary (the left-hand-side angle boundary), where *i* and *j* are related to the four corners. Let *R*_1_ and *R*_2_ be the intersections between the initial sample area and the RAI. For this typical scenario, the intersections among the initial sample area, the right-hand-side angle boundary, the left-hand-side angle boundary, and the RAI (*i.e.*, *r*_11_, *r*_12_, *r*_23_, *r*_24_, *R*_1_, and *R*_2_) are mostly located within the transmission range of a reference node (Figure 5(bottom)). Referring to Figure 5(top), the intersections between the initial sample area and the communication coverage of the reference node can be regarded as new corners of the refined sample area (*i.e.*, (2)′ ← (2), (3)′ ← (3), and (4)′ ← (4)) in Figure 5(bottom). Let *A_ref_* be the communication coverage of the reference node. Thus, the final sample area is the intersection among *A_ref_*, the refined sample area defined by blue lines and our corners: (1), (2)′, (3)′, and (4)′ ordered clockwise, and the RAI with a threshold value of *θ_th_*.

**Figure 6.**
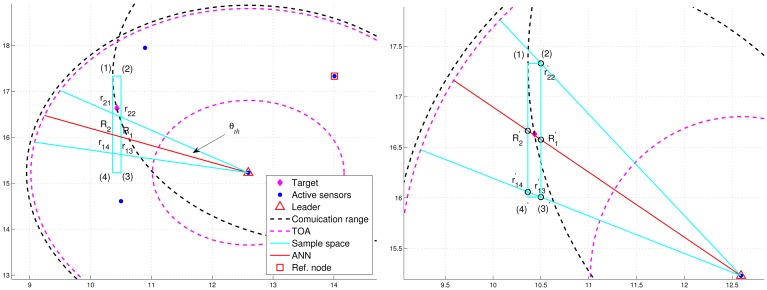
The deviation of RAI from the target (**left**); the updated RAI (**right**).

**Figure 7.**
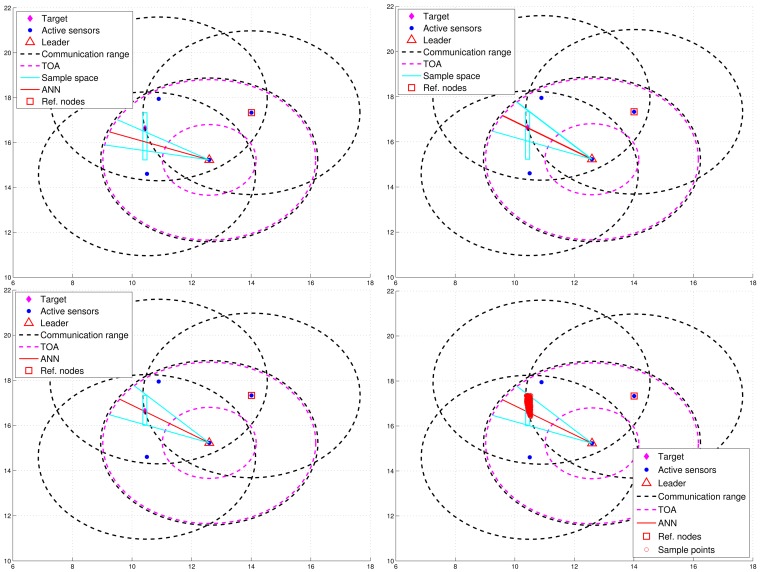
The original RAI (**top left**); the updated RAI (**top right**); the refined sample area (**bottom left**); the final sample area for particle filtering (**bottom right**).

However, Figure 6(left) shows that the deviation of RAI from the target direction and improperly selected threshold values of *θ_th_* (e.g., too small values of *θ_th_*) may lead to the exclusion of possible target locations during the refinement process, which results in an even worse estimate compared with the one without applying the neural networking model. Therefore, in order to avoid the estimation error caused by this scenario, the adjustment principle is to jointly consider the communication coverage of a reference node and the locations of the intersections (*i.e.*, *P*_*r*_1*i*__, *P*_*r*_2*j*__, and *P_R_k__*). Accordingly, if none of the intersection locations is within the communication coverage of the reference node, the updated RAI may be rotated towards the reference node since the original RAI is highly deviated from the target direction. That is, referring to Figure 6(left) and Figure 6(right), if (*P*_*r*_1*i*__ ∉ *A_ref_*, *P*_*r*__2*j*_ ∉ *A_ref_*, and *P_R_k__* ∉ *A_ref_*), the angle information may be updated by $\left(r_{13}^{\prime }\leftarrow R_{1},r_{14}^{\prime }\leftarrow R_{2},\text{and}\;r_{22}^{\prime }\leftarrow r_{22}\right)$. Hence, the left-hand-side angle boundary is replaced by the original RAI and the right-hand-side angle boundary is replaced by the line between the position of the leader node and the corner (2). Afterwards, the intersection of the refined sample area and *A_ref_* forms the final sample area, which generates more effective samples for particle filtering and target positioning. Figure 7 shows an example of angle adjustment for position estimation refinement, which consists of four stages: the original RAI, the updated RAI, the refined sample area, and the final sample area for particle filtering.

#### 3.3.3. Covariance Intersection (CI)

For obtaining global estimates, we adopt covariance intersection to perform data fusion. The CI method of [4] provides the best estimate given the information available, which takes a convex combination of mean and covariance estimates that are represented in information space. Since these typical runs are independent, the general form is

(3) $$P_{cc}^{-1}=\omega_{1}P_{a_{1}a_{1}}^{-1}+\cdots +\omega_{n}P_{a_{n}a_{n}}^{-1}$$

(4) $$P_{cc}^{-1}c=\omega_{1}P_{a_{1}a_{1}}^{-1}a_{1}+\cdots +\omega_{n}P_{a_{n}a_{n}}^{-1}a_{n}$$

where $\sum_{i=1}^{n}\omega_{i}=1$, *n* > 1, *a_i_* is the estimate of the mean from available information, *P_a_i_a_i__* is the estimate of the variance from available information, *c* is the new estimate of the mean, and *P_cc_* is the new estimate of the variance.

#### 3.3.4. Estimation Fusion

The distributed scheme is executed in two steps: (1) Group Estimation: local decisions are performed; (2) Estimation Fusion: a fusion rule is applied to combine the posterior density of the estimation from each member of the cooperative group in the leader sensor. Since the weight reflects the significance attached to the estimate, the next issue is to determine the weigh *ω_i_* for each estimate and try to weight out faulty estimates. One strategy for choosing *ω_i_* is to use the utility measure. Since the utility of a sensor measurement is a function of the geometric location of the target, here we consider the Mahalanobis measure [28]. Hence, with respect to a neighboring system estimate characterized by the mean *μ*_*m*ℓ_ and covariance Σ, the utility function for sensor *m* is defined as the geometric measure

(5) $${\text{U}}_{m\ell }={\left(\mu_{m0}-\mu_{m\ell }\right)}^{T}\Sigma^{-1}(\mu_{m0}-\mu_{m\ell })$$

where *μ_m_*_0_ is the local estimated target position of sensor *m* and ℓ refers to a neighboring system estimate. In order to arrive at a consensus, the utility measure 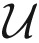
_*m*ℓ_ can be shown to be 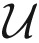
_*m*ℓ_ ≤ 1 [29]. Given the utility measure, two estimates can be allowed to be compared in a common framework and measure how much they differ |*μ_m_*_0_ − *μ*_*m*ℓ_|. Accordingly, the weights for the CI method in (3) are given by

(6) $$\omega_{\ell }=\frac{\frac{1}{{\text{U}}_{m\ell }}}{\sum_{k\in U_{s}}\frac{1}{{\text{U}}_{mk}}}$$

where *U_s_* is the index set of the neighboring estimates that pass the utility test. Otherwise, *ω*_ℓ_ is set to be zero. Notice that in this work *m* may refer to a tasking leader and ℓ may refer to a sub-cluster member.

### 3.4. Phase IV: Sub-Cluster Member Reselection and Leader Handoff

This phase performs the sub-cluster member reselection and leader handoff task, which aims to maintain tracking stability. The conditions for initiating the leader handoff procedure are:

- The distance between the reference location of the sub-cluster member *P_i_* (∀*i* ∈ *M*) and the fused target position estimate *f*(*j*) exceeds the handoff threshold value at time step *j*. That is, *d*(*P_i_*, *f*(*j*)) > ℜ, ∀*i* ∈ *M*.
- Due to the movement of the target, the number of *Position* messages or expected active sensors are less than the desired value. That is, *N_P_* < *n* or *N_E_* < *n*.

**Figure 8.**
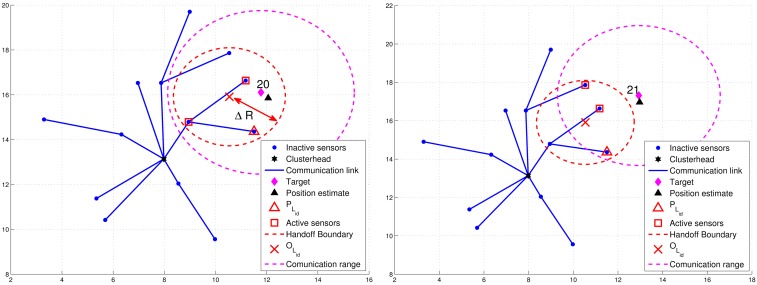
An example of adaptively updating the sub-cluster members with Δ*R.*

**Figure 9.**
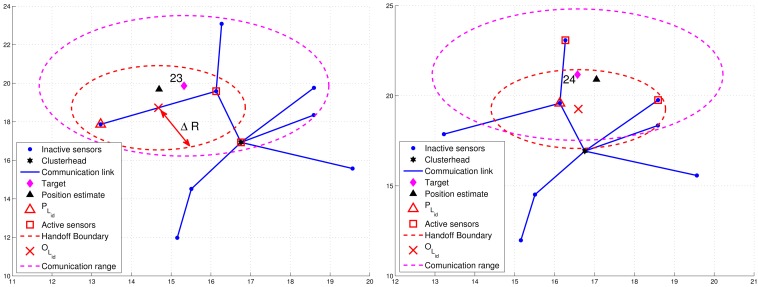
An example of leader handoff procedure.

For condition 1, ℜ = *β* · *R*, where *R* is the radio transmission range and 0 < *β* < 1. For condition 2, a threshold value Δ*R* with Δ*R* = *δ* · *R* (0 < *δ* < 1) for dynamically measuring the number of valid sub-cluster members is applied. Denote *P_L_id__* as the location of leader *L_id_* and *B_L_id__* as the leader handoff boundary (the red dashed line in Figures 8 and 9) of leader *L_id_*, which is a circle of radius Δ*R* centered at *O_L_id__*. Note that *O_L_id__* is the center of gravity of the bounding box, which is derived by the estimated target position, the positions of the sub-cluster members, and the bounding-box algorithm [24]. Thus, *N_P_* is the number of estimates from the sub-cluster members and *N_E_* can be computed as the number of active sensors which satisfy *d*(*O_L_id__*, *P_i_*) ≤ Δ*R*, where *P_i_* is the location of active sensor *i*.

Figure 8 presents an example of adaptively updating the sub-cluster members with Δ*R*. Observe that at time step 20, two sub-cluster members are with tracking responsibility (Figure 8(left)). However, at time step 21, one sub-cluster member becomes an inactive sensor due to the target movement (Figure 8(right)). Thus, the leader will assign an active sensor which is located inside *B_L_id__* to become a new sub-cluster member. If any of the above conditions holds, the leader and target will sequentially broadcast a *Handoff* message with *L_id_* = 0 to trigger a leader reselection process as depicted in Phases I and II. Figure 9 shows an example of leader handoff procedure from time step 23 to time step 24. In Figure 9(left), a handoff procedure is triggered by condition 2. Afterwards, as shown in Figure 9(right), a new leader and its associated sub-cluster members are formed.

Due to the cluster-based network topology, the handoff schemes can be further divided into two categories: (1) intra-cluster leader handoff and (2) inter-cluster leader handoff. Since the clusterhead collects the supplementary estimates and receives the estimate from the leader, it may monitor the *d*(*P_i_*, *f*(*j*)), *N_P_*, and *N_E_*. If condition 1 holds, then an intra-cluster leader handoff is performed and the clusterhead may assign a cluster member to be a new leader. Otherwise, an inter-cluster leader handoff is triggered and the operations move to Phase I and II. The procedures of the TCAT model for cooperative target tracking are detailed in Table 2. Note that *I_Ni_* is the index set of vicinal sensors of sensor *i*, *T_j_* is the true position of target at time step *j*, and *C_i_* is the set of cluster members of sensor *i*.

## 4. Analysis of Positioning Accuracy

Referring to [30], evaluating the computation process and the significance of approximate accuracy is an important step in deriving either exact or approximate solutions for the localization problem. This section presents an estimation-theoretic analysis of the proposed measurement mechanisms to assess the achievable localization accuracy with Cramer–Rao lower bound (CRLB) for joint TOA/AOA estimation.

The measurements at the reference sensor can be modeled as

(7) $$\hat{\tau }=\tau +\delta_{\tau }$$

(8) $$\hat{\phi }=\phi +\delta_{\phi }$$

where *τ* is the true propagation time and *ϕ* is the true angle information. Note that *δ_τ_* and *δ_ϕ_* are uncorrelated Gaussian noises with the distributions $\delta_{\tau }\sim \text{N}(0,\sigma_{\tau }^{2})$ and $\delta_{\phi }\sim \text{N}(0,\sigma_{\phi }^{2})$. Assuming that the direct path exists between the sensor and the target, the estimated target position is given by

(9) $$\hat{x}=x_{s}+v\hat{\tau }\cos(\hat{\phi })=x_{s}+\hat{r}\cos(\hat{\phi })$$

(10) $$\hat{y}=y_{s}+v\hat{\tau }\sin(\hat{\phi })=y_{s}+\hat{r}\sin(\hat{\phi })$$

where *r̂* is the distance measurement (*i.e*., *r̂* = *vτ̂* = *r* + *vδ_τ_*), (*x_s_*, *y_s_*) is the true position of the sensor and *v* is the speed of signal. Assuming *δ_τ_* and *δ_φ_* are sufficiently small, the variance of the position estimation *p̂* is approximated by

(11) $$\sigma_{p}^{2}\approx v^{2}\sigma_{\tau }^{2}+d^{2}\sigma_{\phi }^{2}=\sigma_{r}^{2}+d^{2}\sigma_{\phi }^{2}$$

Given the above assumptions [31], the CRLB with a single sensor is derived as follows. The probability density function of g = [*r̂*, *ϕ̂*] is

(12) $$f(g;x,y)=\frac{1}{\sqrt{2\pi \sigma_{r}^{2}}}\cdot \exp\left[-\frac{1}{2\sigma_{r}^{2}}{\left(\hat{r}-d\right)}^{2}\right]\cdot \frac{1}{\sqrt{2\pi \sigma_{\phi }^{2}}}\cdot \exp\left[-\frac{1}{2\sigma_{\phi }^{2}}{\left(\hat{\phi }-\arctan\left(\frac{y-y_{s}}{x-x_{s}}\right)\right)}^{2}\right]$$

Thus, the Fisher information matrix yields

(13) $$I({\text{x}}^{\left(t\right)})=\left[\begin{array}{cc} \frac{\cos^{2}(\phi )}{\sigma_{r}^{2}}+\frac{\sin^{2}(\phi )}{d^{2}\sigma_{\phi }^{2}} & \frac{\sin(2\phi )}{2}\left[\frac{1}{\sigma_{r}^{2}}-\frac{1}{d^{2}\sigma_{\phi }^{2}}\right]\\ \frac{\sin(2\phi )}{2}\left[\frac{1}{\sigma_{r}^{2}}-\frac{1}{d^{2}\sigma_{\phi }^{2}}\right] & \frac{\sin^{2}(\phi )}{\sigma_{r}^{2}}+\frac{\cos^{2}(\phi )}{d^{2}\sigma_{\phi }^{2}} \end{array}\right]$$

and the CRLB can then be written as

(14) $$\text{V ar}({\text{x}}^{\left(t\right)})\geq I^{-1}({\text{x}}^{\left(t\right)})=\left[\begin{array}{cc} I_{1,1}^{\prime } & I_{1,2}^{\prime }\\ I_{2,1}^{\prime } & I_{2,2}^{\prime } \end{array}\right]$$

Thus,

(15) $$\begin{array}{cc} \text{V ar}(\tilde{x})\geq I_{1,1}^{\prime }, & \text{V ar}(\tilde{y})\geq I_{2,2}^{\prime } \end{array}$$

Accordingly, the best possible fused estimation for target localization may be determined by applying Equation (15) and the CI method.

## 5. Analysis of Energy Consumption

This section considers the energy consumption of the proposed scheme in Section 3. It is often the case that inter-sensor communication costs are greater by orders of magnitude than local computation and sensing costs with respect to energy expenditures [22,32,33]. Therefore, the computational cost in a wireless sensor network is usually neglected compared to the communication cost. In addition, the problem of energy waste in idle listening mode can be solved by periodic listen and sleep. Moreover, based on the data sheet of CC2420 [34], the energy consumption of receive mode is about 59.1 mW, the energy consumption of transmit mode is about 52.2 mW, and the energy consumption of idle listening mode is about 1.2 mW. Accordingly, we focus on the comparison of power consumption for communication with the scheme of [5]. The total power requirements include both the power required to transmit messages and the power required to receive (or process) messages. Suppose that the energy needed to transmit for sensors with omnidirectional antennas is *E_T_*, which depends on the transmitting range *R*, and the energy needed to receive is *E_R_*.

When the target broadcasts a message with *L_id_* = 0, its neighboring sensor, say sensor *i*, becomes an active sensor and broadcasts a *Hello* message with a random waiting time ${\text{LWT}}_{i}^{\left(j\right)}$ for being a task leader at time step *j*. As the active sensor *i* claims to be a leader, the *L_id_* is updated and broadcasted from target. As a result, the number of transmissions and receptions for tasking leader selection (Phase I) are

(16) $${\text{S}}_{T}^{c}(j)=1+N_{t}(j)$$

(17) $${\text{S}}_{R}^{c}(j)=\sum_{i\in (I_{A}^{\left(j\right)}\cup L_{id}^{\left(j\right)})}\left|I_{N_{i}}|+2N_{t}(j\right)$$

where $L_{id}^{\left(j\right)}$ is a leader ID at time step *j*, *I_N_i__* is the index set of vicinal sensors of sensor *i*, and *N_t_*(*j*) is the number of transmissions of vicinal sensors of the target at time step j.

Afterward, the active sub-cluster members of the leader are selected according to the extra backoff time *BT_m_*, which transmit the *Position* messages to the leader. Thus, the number of transmissions yields the sub-cluster size $\left|M(j)\cap I_{N_{L_{id}^{\left(j\right)}}}\right|$ and the number of receptions is $\left|I_{N_{i}}|(\forall i\in M(j)\cap I_{N_{L_{id}^{\left(j\right)}}}\right)$, where the *M*(*j*) is the set of sub-cluster members at time step *j*. Since the fused estimate *f*(*j*) is routed to the clusterhead in a multi-hop manner, the number of transmissions is ${\text{N}}_{i}^{H}(\forall i\in {\left(I_{A}^{\left(j\right)}\cap I_{N_{L_{id}^{\left(j\right)}}}\right)}^{c})$, where ${\text{N}}_{i}^{H}$ is number of hops for sensor *i* to perform estimation reporting. Finally, the clusterhead disseminates *f*(*j*) to the base station. Furthermore, if the clusterhead receives a message from the leader for incorporating supplementary estimates, it may assign a desired number of supplementary members to be supplementary sub-cluster members for the tracking task. Therefore, the number of transmissions and receptions for selecting tasking members (Phase II) and delivering the position estimate (Phase III) are

(18) $${\text{S}}_{T}^{p}(j)=\left|M(j)\cap I_{N_{L_{id}^{\left(j\right)}}}\right|+\sum_{i\in {\left(I_{A}^{\left(j\right)}\cap I_{N_{L_{id}^{\left(j\right)}}}\right)}^{c})}{\text{N}}_{i}^{H}+\sum_{i\in M\prime }{\text{N}}_{i}^{H}+1$$

(19) $${\text{S}}_{R}^{p}(j)=\sum_{i\in (M(j)\cap I_{N_{L_{id}^{\left(j\right)}}})}\left|I_{N_{i}}\right|+(\sum_{i\in {\left(I_{A}^{\left(j\right)}\cap I_{N_{L_{id}^{\left(j\right)}}}\right)}^{c}}+\sum_{i\in M\prime })\sum_{h=1}^{{\text{N}}_{i}^{H}}N_{I_{R_{i}}^{\left(h\right)}}$$

where $M\prime =\{i:{\text{argmin}}_{i}\sum {\text{N}}_{i}^{H},i\in M(j)\cap {\left(I_{A}^{\left(j\right)}\cap (I_{N_{L_{id}^{\left(j\right)}}}\cup L_{id}^{\left(j\right)})\right)}^{c}\}$ and $I_{R_{i}}^{\left(h\right)}$ is index set of relay ID for sensor *i* at hop *h*.

As characterized in Phase IV, since the clusterhead has the capability of updating the sub-cluster members, a cluster member, say sensor *i*, may become an active supplementary sub-cluster member or leader when receiving the message, which contains the IDs of new supplementary sub-cluster member or the leader (*i.e*., (*i* ∩ *M̅*(*j*) ≠ Ø) or leader ID), and then joint the tracking task. Note that *M̅*(*j*) is the index set of new supplementary sub-cluster members. Therefore, we obtain

(20) $${\text{S}}_{T}^{f}(j)=\sum_{i\in M\prime \prime }{\text{N}}_{i}^{H}+1$$

(21) $${\text{S}}_{R}^{f}(j)=N_{CH(j)}+\sum_{i\in M\prime \prime }\sum_{h=1}^{{\text{N}}_{i}^{H}}N_{I_{R_{i}}^{\left(h\right)}}$$

where $M\prime \prime =\{i:{\text{argmin}}_{i}\sum {\text{N}}_{i}^{H},i\in {\left(\bar{M}(j)\cap I_{N_{CH(j)}}\right)}^{c}\cup {\left(L_{id}^{\left(j+1\right)}\cap L_{id}^{\left(j\right)}\right)}^{c}\}$ and ${\text{S}}_{T}^{f}(j)$ and ${\text{S}}_{R}^{f}(j)$ are the number of transmissions and receptions for leader handoff, respectively. Nonetheless, when the leader ID is zero $\left(i.e.,L_{id}^{\left(j+1\right)}=0\right)$, the sub-cluster members are assigned to be inactive sensors and the procedure of selecting a new sub-cluster will be triggered. Accordingly, the total energy consumption of transmission and reception for tracking target is $E^{\text{TCAT}}=\sum_{j=1}\left(E_{T}\cdot ({\text{S}}_{T}^{c}(j)+{\text{S}}_{T}^{p}(j)+{\text{S}}_{T}^{f}(j))+E_{R}\cdot ({\text{S}}_{R}^{c}(j)+{\text{S}}_{R}^{p}(j)+{\text{S}}_{R}^{f}(j))\right)$.

## 6. Simulation

To evaluate the performance of the proposed approach, assume that the target moves within the *x* − *y* sensing field according to the standard second-order model [2]

(22) $$X_{k}=\Phi X_{k-1}+\Gamma {\text{w}}_{k}$$

over a four-dimensional state space, where $X_{k}={\left(x,\dot{x},y,\dot{y}\right)}_{k}^{T}$, ${\text{w}}_{k}={\left(w_{x},w_{y}\right)}_{k}^{T}$, an uncorrelated Gaussian diffusion term describing the uncertainty,

$$\Phi =\left(\begin{array}{cccc} 1 & 1 & 0 & 0\\ 0 & 1 & 0 & 0\\ 0 & 0 & 1 & 1\\ 0 & 0 & 0 & 1 \end{array}\right),\text{and}\;\Gamma =\left(\begin{array}{cc} 0.5 & 0\\ 1 & 0\\ 0 & 0.5\\ 0 & 1 \end{array}\right)$$

Here *x* and *y* denote the Cartesian coordinate of the target. The noisy measurement is given by

(23) $$z_{k}=\tan^{-1}(y_{k}/x_{k})+v_{k}$$

where the measurement noise, *v_k_*, is a zero mean Gaussian white noise process with a finite variance $\sigma_{\theta }^{2}$. Before measurements are taken at *k* = 1, the initial state vector is assumed to be a Gaussian distribution with known mean *x̄*_1_, and covariance

$$M_{1}=\left(\begin{array}{cccc} \sigma_{1}^{2} & 0 & 0 & 0\\ 0 & \sigma_{2}^{2} & 0 & 0\\ 0 & 0 & \sigma_{3}^{2} & 0\\ 0 & 0 & 0 & \sigma_{4}^{2} \end{array}\right)$$

The target trajectory and measurements are generated based on Equations (22) and (23) with the parameter values: the covariance of the system noise, *Q* = *qI*_2_, where *I*_2_ is the 2 × 2 identity matrix, $\sqrt{q}=0.001$. The initial state of the target is *x*_1_ = (0.0, 0.1, 0.0, 0.05)*^T^*. The prior distribution parameters are set to *x̄*_1_ = (0.0, 0.0, 0.4, 0.05)*^T^* and *σ*_1_ = 0.5, *σ*_2_ = 0.001, *σ*_3_ = 0.05 and *σ*_1_ = 0.01. The target position estimate is conducted with *N_PF_* = 1000 samples, the ANN model, and the CI method for 25 time steps.

**Figure 10.**
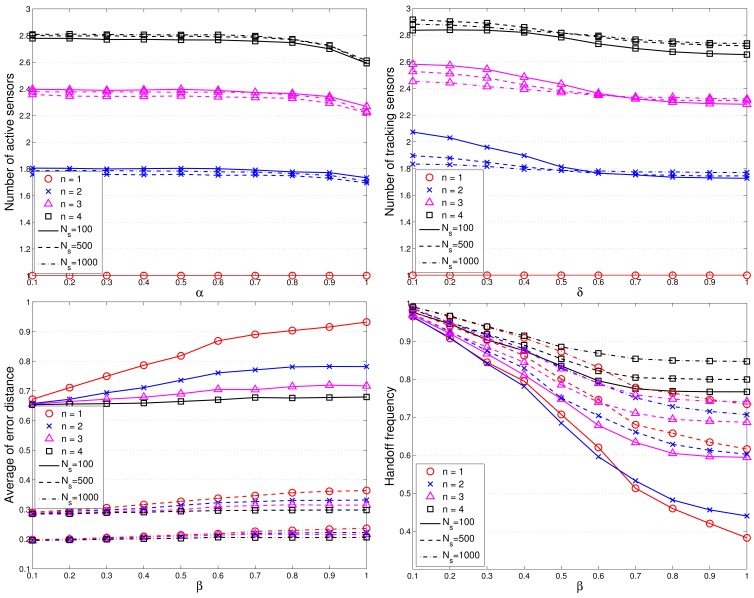
The target localization error with *β* = *δ* = 0.5 and varying the values of *α* (**top left**); The target localization error with *α* = *β* = 0.5 and varying the values of *δ* (**top right**); The target localization error with *α* = *β* = 0.5 and varying the values of *β* (**bottom left**); The leader handoff frequency with *α* = *δ* = 0.5 and varying the values of *β* (**bottom right**).

Figure 10 depicts the system performance (e.g., the average positioning error and the leader handoff frequency) with various values of parameters (*α*, *β*, *δ*, sub-cluster size, and network density), for cooperative target tracking. Observe that for parameter *β*, there is a tradeoff between localization error and leader handoff frequency since a larger value of *β* (*i.e*., a larger handoff threshold value) may lead to a lower leader handoff frequency and may result in a lower positioning accuracy. Without loss of generality, we investigate the typical performance of the TCAT in a network with random uniform deployment of *N_S_* sensors given *α* = *β* = *δ* = 0.5, *C* = *D* = 1, and the standard deviation of angle information *σ_θ_* = 0.5 radian. Note that the entire experiments are conducted in a square with side length *L* = 30 unit length and transmission range $R=L\sqrt{{\text{log}}_{10}(L)/N_{S}}$ [35].

### 6.1. Performance of Neural Networking Model

**Figure 11.**
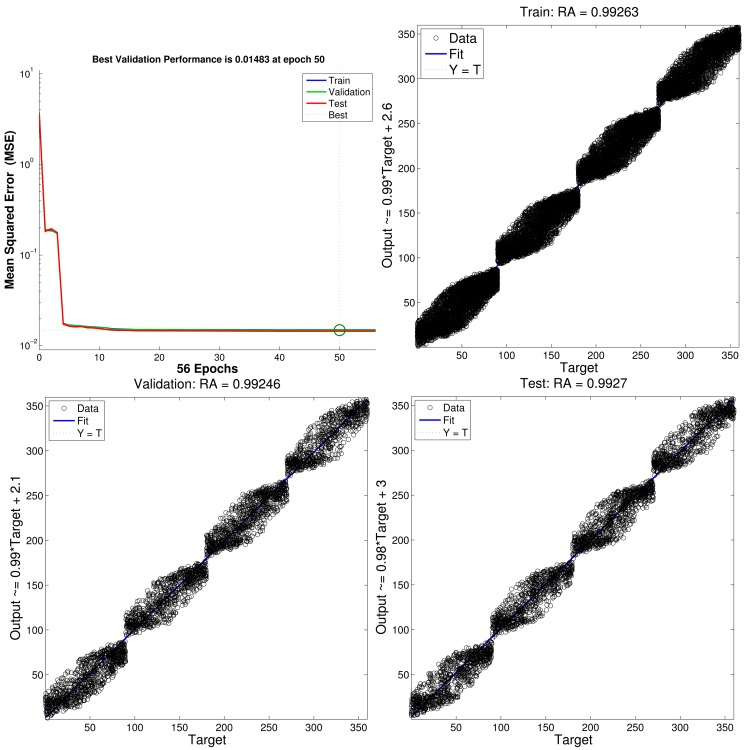
The performance analysis of the network (**top left**); the regression analysis between the network response and the corresponding targets: the training case (**top right**), the validation case (**bottom left**) and the test case (**bottom right**).

For the CFBP model, Figure 11 depicts the learning and regression analysis of the network. Figure 11 (top left) shows that the network is learning since the mean squared error of the network is decreasing to a smaller value and the training of CFBP network is stopped before overfitting. The 22,804 input and target vectors are randomly divided into three sets. 15,962 vectors are used to train the network. Of these vectors, 3,421 are used to validate how well the network generalized. Finally, the last 3,421 vectors provide an independent test of network generalization to data that the network has never seen. Moreover, regression analysis is employed as post-training analysis between the network response and the corresponding targets and three parameters are returned to evaluate the performance. The first two parameters, slope and y-intercept of the best linear regression relate targets to network outputs. If the outputs exactly equal to targets, the slope and y-intercept would be 1 and 0, respectively. For the training case, slope = 0.99 and y-intercept = 2.6. For the validation case, slope = 0.99 and y-intercept = 2.1. For the test case, slope = 0.98 and y-intercept = 3. The third parameter is correlation coefficient between the outputs and targets. When the correlation coefficient is equal to 1, then there is perfect correlation between targets and outputs. In this study, the correlation coefficients of the regression analysis is about *RA* = 0.99 as shown in Figure 11, which therefore illustrates a good fit.

### 6.2. Target Localization Error

In order to further explore the effectiveness of the proposed refinement scheme, four different information measurement scenarios are considered: (1) AOA information only; (2) AOA with neural network (ANN) refinement; (3) Joint TOA/AOA information only; (4) Joint TOA/AOA with ANN refinement.

#### 6.2.1. AOA with/without ANN Refinement

**Figure 12.**
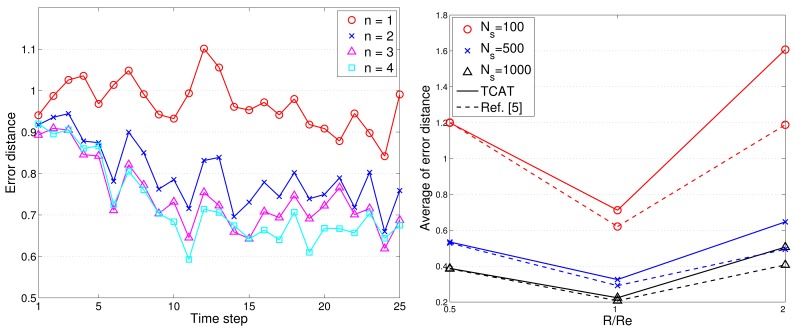
The target localization error (**left**); the estimation error with *n* = 2 and various ratios of *R*/*R_e_* (**right**).

To assess the tracking accuracy, the root mean square error is used for comparing the tracking accuracy of the distributed TCAT with that of [5]. Referring to the network topology and the target movement in Figure 2(left), we vary the number of sub-cluster members from 1 to 4. Figure 12(left) shows the accuracy of the position estimate. The performance improves along with the number of sub-cluster size *n*. However, the improvement is not significant (especially when the number *n* is greater than 2). This suggests that even a low number of sub-cluster members can also achieve good estimation accuracy.

As illustrated in Figure 12(left), the radio transmission range *R* is assumed to be the same as the target detection range *R_e_*. Here the effect of varying target detection ranges on the performance is investigated with changing the ratio of the radio transmission range to target detection range. Figure 12(right) depicts that when the ratio is greater than one (*i.e*., *R*/*R_e_* > 1), the sensors may fail to detect most target events and a larger network density may be required to detect the target of small signal magnitude and to suppress the estimation error. However, the estimation error decreases dramatically when the ratio *R*/*R_e_* ≤ 1 due to sufficient detection coverage.

**Figure 13.**
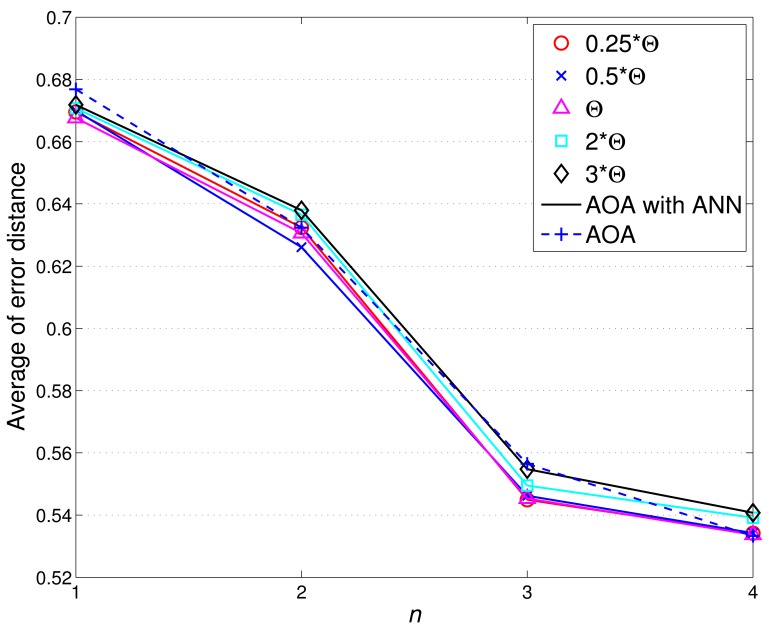
The comparison of target localization error with/without ANN refinement.

Notice that the above performance evaluation is based on AOA information only without executing estimation refinement. Figure 13 shows the comparison of target localization error with/without ANN refinement. With a moderate value of *θ_th_* (e.g., 0.25Θ ≤ *θ_th_* ≤ Θ with Θ = 9.98 degree), the proper refined sample area results in an improvement of estimation accuracy. However, loose NN-based angle information (e.g., Θ ≤ *θ_th_* ≤ 3Θ) may generate a broader sample area, which degrades the estimation performance.

#### 6.2.2. TOA/AOA with/without ANN Refinement

**Figure 14.**
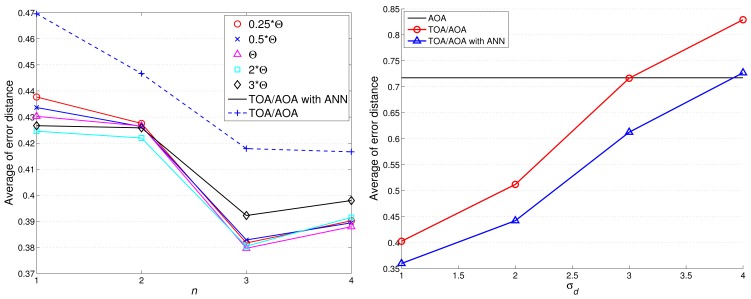
The comparison of target localization error with/without ANN refinement (**left**); the performance comparison of TCAT with joint AOA/TOA and TCAT with AOA only with varying the deviation of TOA measurement (**right**).

Compared with Figure 13, Figure 14(left) shows that the average localization error is significantly suppressed by applying the TOA information. Considering *n* = 2 and *θ_th_* = 9.98 degree, the average localization error of TCAT using AOA/TOA information with ANN refinement is about 36% less than that of TCAT using AOA information with ANN refinement. Nonetheless, as the deviation of TOA measurement increases, the localization performance of TCAT with joint TOA/AOA is approaching to that of TCAT with AOA only (Figure 14(right)). This is attributed to the fact that a noisy TOA measurement may lead to a broader sample area (*i.e*., a lower particle density) for Bayesian filtering, which enlarges the localization error.

**Figure 15.**
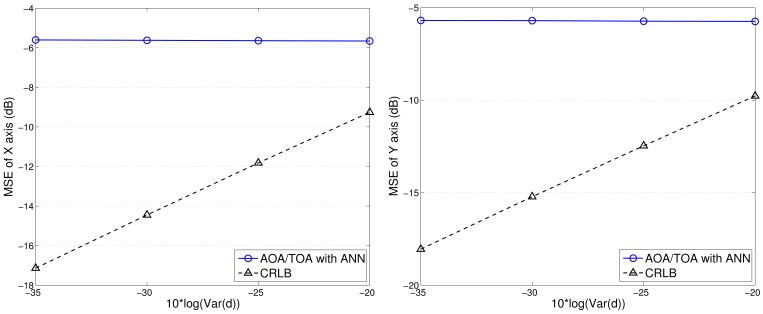
The performance comparison of TCAT with joint AOA/TOA and CRLB: X-axis (**left**) and Y-axis (**right**).

Given the variance of angle estimation *σ_θ_* = 0.5 and with varying the uncertainty of distance estimation *σ_d_*, here we consider the best achievable performance and the tracking performance with TCAT using TOA/AOA information. Notice that the CRLB and the performance of the proposed method tend to merge together with an increasing measurement uncertainty *σ_d_*. Due to a small sample size for particle filtering and the limited capability of a sensor, as shown in Figure 15, fundamental problems when locating mobile target in a network are to estimate the distance between the reference sensors and the target and to determine the angle of arrival of the signals since accurate location estimates highly rely on precise TOA/AOA measurements and the processing capability of a sensor node.

### 6.3. Protocol Characteristics

**Figure 16.**
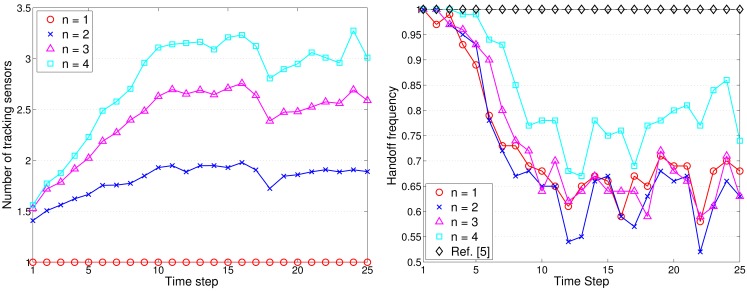
The typical runs of sub-cluster formation (**left**); the frequency of leader handoff (**right**).

Figure 16(left) shows the typical runs of sub-cluster formation with *N_S_* = 100 and *R*/*R_e_* = 1. Notice that the TCAT effectively organizes the sensors into tracking groups. Referring to Figures 12 (left) and Figure 16(right), observe that compared with the protocol in [5], the TCAT has a lower leader handoff frequency and there is no significant performance degradation during the leader handoff period. Moreover, considering different sub-cluster size *n* and a network with random uniform deployment, Figures 12(left) suggests that compared with the TCAT with *n* = 2, the TCAT with a smaller value of *n* (e.g., *n* = 1) with respect to handoff condition 1 and the TCAT with a larger value of *n* (e.g., *n* = 3, 4) with respect to handoff condition 2 lead to a higher frequency of inter-cluster handoff. Thus, the TCAT with *n* = 2 may provide flexibility and robustness for distributed sensor scheduling management.

### 6.4. Network Energy Consumption

The simulation is performed with different density of nodes, considering the number of messages transmitted and received involved in clustering and target localization. In order to evaluate the network performance, several models for measuring the energy dissipation per transmitted bit have been proposed [36,37]. Here, the energy model presented in [36] is applied to describe the energy dissipation. Assume the hardware energy dissipation is as follows [36]:

(24) $$E_{Tx}=\{\begin{array}{cc} tE_{\text{elec}}+t\varepsilon_{fs}d^{2}, & d<d_{o}\\ tE_{\text{elec}}+t\varepsilon_{mp}d^{4}, & d\geq d_{o} \end{array}$$

(25) $$E_{Rx}=tE_{\text{elec}}$$

where *E_Tx_* and *E_Rx_* are energy consumption of a transmitter and a receiver, respectively, *t* is the data packet size, *E_elec_* denotes the energy consumption of the electronic circuitry, *ε_fs_* and *ε_mp_* depend on distance *d* between the transmitter and the receiver for maintaining an acceptable bit-error rate, and *d_o_* is a threshold of the transmission. The values of simulation parameters are detailed in Table 3 [36].

**Figure 17.**
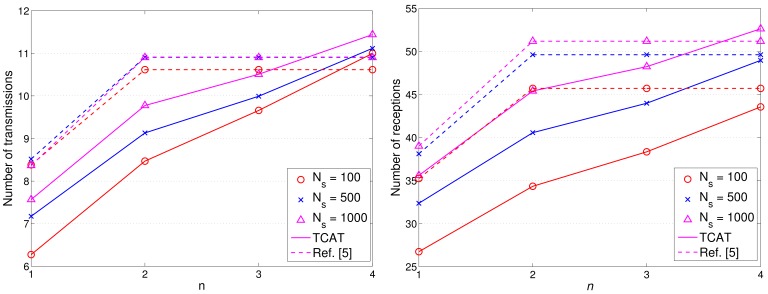
The number of transmission/reception: the communication cost.

**Figure 18.**
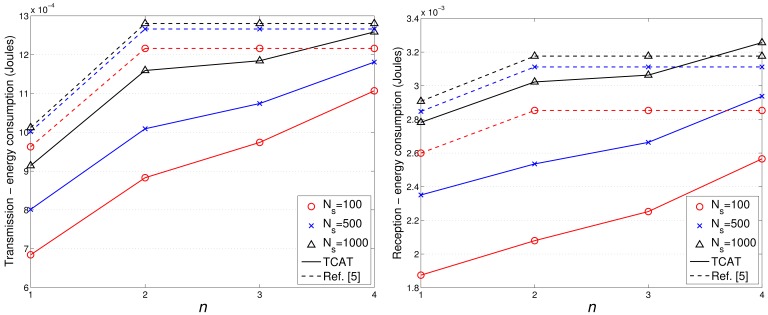
The comparison of accumulated network energy consumption.

Figures 17 and 18 show the accumulated energy consumption comparison between the TCAT scheme and the method in [5]. Observe that the number of transmissions/receptions grows nearly linearly as the tracking sub-cluster size increases. Referring to Figure 17, compared with a network with *N_S_* = 100 and 1 ≤ *n* ≤ 3, a network with a larger scale (e.g., *N_S_* = 500, 1000) may have a larger cluster size, which may lead to a higher number of transmission/reception for data gathering at each round and result in a faster network resource depletion. Nonetheless, the performance of TCAT is still superior to that of the approach in [5].

Given *n* = 2, *N_S_* = 100, and *R*/*R_e_* = 1, Figure 12(right) shows that the tracking accuracy of TCAT is comparable to that of [5]. Moreover, as depicted in Figure 18(left), the energy consumption of transmissions with TCAT is about 26.4% less than that of [5] and in Figure 18(right) the energy consumption of receptions with TCAT is about 25% less than that of [5], which implies that the scheme in [5] may lead to a fast network energy depletion. Accordingly, the TCAT provides better network service characteristics compared to the protocol of [5].

Observe that in Figures 12 and 18, although the performance of TCAT with *n* = 1 leads to a lower network energy consumption, compared with those of TCAT with *n* = 2 ∼ 4, it results in a larger target localization error. Moreover, due to high correlation of sensing data in time and spatial domains, the sub-cluster with *n* > 2 members may lead to undesired sensing redundancy. Therefore, considering the trade-off between performance and network energy consumption, the TCAT with *n* = 2 may be a good choice for the tracking task.

## 7. Conclusions

Because of the resource-constrained sensors, feasible wireless sensor-based tracking systems require more breakthroughs in terms of network architecture, system design, and data processing techniques. In order to achieve good tracking quality, the number of sensors chosen for target positioning may be dynamically adjusted based on the available target and sensor information. Thus, incorporating the target motion information into cooperative positioning schemes with multiple sensors may be a good strategy to improve the estimation accuracy. Future plans will involve generalizing the method to implement a prototype of the tracking system, evaluate the merits of different cooperative schemes, explore the characteristics of target mobility model, and further examine the impact of target motion information on cooperative estimation performance.

## Figures and Tables

**Table 1.** The Particle Filtering Methodology.

*Initialization*: Generate a set of random samples *x_k_*(*i*), *i* = 1, 2, …, *N_PF_* from the prior density at time *k* = 0. Each sample of the state vector is a “particle”. *Prediction*: Each random sample is passed through the state equation to obtain samples from the prior density at time *k* + 1. Thus $${\hat{x}}_{k+1}(i)=\Phi x_{k}(i)+\Gamma \lambda_{k}(i)$$ where *λ_k_*(*i*) is a sample drawn from the probability density function of the system noise, Φ is related to the mobility model, and Γ is an identity matrix. *Measurement Update*: The weights of the likelihood function *p*(*z_k_*_+1_∣*x̂_k_*_+1_(*i*)) are updated for each sample in the random set *i* = 1, 2, …, *N_PF_* and the normalized weights are $$\xi_{k+1}(i)=\frac{p(z_{k+1}|{\hat{x}}_{k+1}(i))}{\sum_{j=1}^{N_{PF}}p(z_{k+1}|{\hat{x}}_{k+1}(j))}$$ for each sample. *Re-sampling*: Take *N_PF_* samples with replacement from the random sample set *x̂_k_*_+1_(*i*), *i* = 1, 2, …, *N_PF_*, to generate the new sample set *x_k_*_+1_(*i*). *Position*: The best single estimate of the position is the mean of *x_k_*_+1_(*i*), $\bar{x_{k}}$.

**Table 2.** Procedures of the TCAT model for target tracking.

Target broadcasts a message with *L_id_* = 0. Determine the active sensors $I_{A}^{\left(j\right)}$ and the leader *L_id_* at time step *j*. $L_{id}={\text{argmin}}_{i}LWT_{i}^{\left(j\right)},i\in I_{A}^{\left(j\right)};M=L_{id}$. $BT_{i}=LWT_{i}+bf_{i},i\in I_{CA};I_{CA}=I_{A}^{\left(j\right)}\cap C_{L_{id}}$. Find the sub-cluster members: while(*I_CA_* ≠ Ø) if((*S* = *I_N_L_id___*) == Ø), *S* = (*I_N_L_id___* ∩ *I_CA_*)*^c^*. $\hat{M}={\text{argmin}}_{i}BT_{i}^{\left(j\right)},i\in S$. Send the position estimate to the leader or clusterhead. *M* = *M* ∪ *M̂; S* = (*S* ∩ *M*)*^c^*; *I_CA_* = (*I_CA_* ∩ *M̂*)*^c^*. if(|*M*| == *n*), break. end Estimation Fusion: Leader sends the global fused estimate *f*(*j*) to the clusterhead. The clusterhead disseminates the *f*(*j*) to the base station. Perform leader handoff (renewing sub-cluster members): *N_P_* = |*H_P_*|, *H_P_* = {*i: d*(*T_j_*, *i*) ≤ *R*, *i* ∈ *M*}. if(*N_P_* < *n*), *j* = *j* + 1 and go to Step 1. if(*d*(*P_L_id__*, *f*(*j*)) > ℜ) *M* = (*M* ∩ *L_id_*)*^c^*, *K* = {*i*: argmin*_i_d*(*P_i_*, *f*(*i*)) ≤ ℜ,*i* ∈ *C_L_id__*}, *L_id_* = argmax*_k_N_s_*(*k*), *k* ∈ *K*, if(*L_id_* == Ø), *j* = *j* + 1; go to Step 1. end *I_CA_* = (*C_L_id__* ∩ *M*)*^c^*. *N_E_* = |*H_E_*|, *H_E_* = {*i: d*(*O_L_id__*, *P_i_*) ≤ Δ*R*, *i* ∈ *M*}. while(*N_E_* < *n*) *M̂* = argmin*_i_d*(*O_L_id__*, *P_i_*) ≤ *R*, *i* ∈ *I_CA_*. *N_E_* = *N_E_* + 1; M = *M* ∪ *M̂; I_CA_* = (*I_CA_* ∩ *M̂*)*^c^*. if(*I_CA_* == Ø), break. end if(*N_E_* < *n*), *j* = *j* + 1; go to Step 1. Target broadcasts a message with *L_id_* at time step *j* + 1. Go to Step 4.

**Table 3.** The Values of Simulation Parameters [36].

**Parameter**	**Value**
*E_elec_*	50 nJ/bit
*ε_fs_*	10 pJ/bit/m^2^
*ε_mp_*	0.0013 pJ/bit/m^4^
*d_o_*	Transmission range *R*
Energy for data aggregation	5 nJ/bit/signal
Data packet size *t*	2048 bits
